# *ALKAL1* gene silencing prevents colorectal cancer progression via suppressing Sonic Hedgehog (SHH) signaling pathway

**DOI:** 10.7150/jca.46447

**Published:** 2021-01-01

**Authors:** Shasha Chen, Bin Wang, Xuekun Fu, Yanfang Liang, Xingxing Chai, Ziyu Ye, Ronggang Li, Yaoming He, Gang Kong, Jiachun Lian, Xiangyong Li, Ting Chen, Xin Zhang, Xianxiu Qiu, Xudong Tang, Keyuan Zhou, Bihua Lin, Jincheng Zeng

**Affiliations:** 1 Guangdong Provincial Key Laboratory of Medical Molecular Diagnostics, Dongguan Key Laboratory of Medical Bioactive Molecular Developmental and Translational Research, Guangdong Medical University, Dongguan 523808, China.; 2 Law Sau Fai Institute for Advancing Translational Medicine in Bone and Joint Diseases, School of Chinese Medicine, Hong Kong Baptist University, Hong Kong, China.; 3 Department of Pathology, Dongguan Hospital Affiliated to Medical College of Jinan University, Marina Bay Central Hospital of Dongguan, Dongguan 523905, China.; 4 Laboratory Animal Center, Guangdong Medical University, Zhanjiang, 524023 China.; 5 Department of Pathology, Jiangmen Central Hospital, Affiliated Jiangmen Hospital of Sun Yat-sen University, Jiangmen 529030, China.; 6 Department of Gastrointestinal Surgery, Jiangmen Central Hospital, Affiliated Jiangmen Hospital of Sun Yat-sen University, Jiangmen 529030, China.; 7 Key Laboratory of Medical Bioactive Molecular Research for Department of Education of Guangdong Province, Guangdong Medical University, Dongguan 523808, China.; 8 Collaborative Innovation Center for Antitumor Active Substance Research and Development, Guangdong Medical University, Zhanjiang, Guangdong 524023, China.; 9 Clinical Experimental Center, Jiangmen Central Hospital, Affiliated Jiangmen Hospital of Sun Yat-sen University, Jiangmen, 529030, China.

**Keywords:** ALKAL1, colorectal cancer, SHH signaling pathway, metastasis

## Abstract

Anaplastic lymphoma kinase (ALK) has been described in a range of human cancers and is involved in cancer initiation and progression via activating multiple signaling pathways, such as the PI3K-AKT, CRKL-C3G, MEKK2/3-MEK5-ERK5, JAK-STAT and MAPK signal pathways. Recently ALK and LTK ligand 1 (ALKAL1) also named “augmentor-β” or “FAM150A” is identified as a potent activating ligands for human ALK that bind to the extracellular domain of ALK. However, due to its poor stability, the mechanisms of ALKAL1 underlying the tumor progression in the human cancers including colorectal cancer have not been well documented. Herein, ALKAL1 expression was evaluated by RNA sequencing datasets from The Cancer Genome Atlas (TCGA) of 625 cases colorectal cancer, immunohistochemical analysis of 377 cases colorectal cancer tissues, and Western blotting even Real-time PCR of 10 pairs of colorectal cancer tissues and adjacent normal tissues, as well as 8 colorectal cancer cell lines. Statistical analysis was performed to explore the correlation between ALKAL1 expression and clinicopathological features in colorectal cancer. Univariate and multivariate Cox regression analysis were performed to examine the association between ALKAL1 expression and overall survival. *In vitro* and *in vivo* assays were performed to assess the biological roles of ALKAL1 in colorectal cancer. Gene set enrichment analysis (GSEA), Western blotting and luciferase assays were used to identify the underlying signal pathway involved in the tumor progression role of ALKAL1. As a result, we showed that ALKAL1 was upregulated in colorectal cancer tissues and cell lines. Upregulation of ALKAL1 correlated with tumor malignancy and poor prognosis in colorectal cancer. ALKAL1 silencing inhibited tumorigenesis, metastasis and invasion of colorectal cancer cells, and inhibited SHH signaling pathway, which is essential for ALKAL1 induced migration. Our findings reveal a new mechanism by which ALKAL1 participates in colorectal cancer migration and invasion via activating the SHH signaling pathway.

## Introduction

Colorectal cancer is the third most common malignancy in men and the second most common malignancy in women. The latest global colorectal cancer statistics showed that the incidence is 30 percent or 40 percent higher in men than in women 1. Despite advances in the early diagnosis, 50%-60% of colorectal cancers have metastasized when diagnosed, thus resulting in a poor prognosis. Metastasis is the major cause of death in the colorectal cancer. Our previous results demonstrated that dysregulation of miRNAs (miR-196b-5p) 2, 3, tumor-associated immune cells (cytotoxic lymphocytes) 4, and inflammatory mediator (IL-35, EBI3) 4, 5 are associated with metastasis of colorectal cancer. Recently, several drugs (bevacizumab, cetuximab, aflibercept, regorafenib and panitumumab) have been approved by the U.S. Food and Drug Administration (FDA) for treatment of metastatic colorectal cancer. However, the limitations of these drugs need attention, such as low selectivity, insufficient concentrations in tumor tissue, and systemic toxicity 6. A better understanding of the molecular regulation of colorectal cancer metastasis will help us to provide therapeutic interventions for metastatic colorectal cancer.

The Hedgehog (HH) signaling pathway, also known as Hedgehog-Patched (HH-PTCH), Hedgehog-Gli (HH-GLI) or Hedgehog-Patched-Smoothened (HH-PTCH-SMO), was first identified in the common fruit fly. Hh signaling pathway is an evolutionarily conserved pathway of signal transmission from the cell membrane to the nucleus to involve in normal embryonic development 7, the maintenance of airway epithelial progenitors and pluripotent cells 8, regeneration of the lung and prostate epithelium for tissue repair 9, and various stages of carcinogenesis in different tumors 10. Sonic Hedgehog (SHH), a secreted protein belonging to the HH family, have been reported to involve in endothelial cell growth, cell migration and the formation of new blood vessels 11. Deregulation of the SHH signaling pathway has been implicated in lymphoma such as small lymphocytic lymphoma, plasma cell myeloma, mantle cell lymphoma, diffuse large B-cell lymphoma, chronic myelogenous leukemia, acute leukemias and anaplastic lymphoma kinase (ALK) - positive anaplastic large cell lymphoma, as well as sporadic cancers such as gastric cancer, basal cell carcinoma, medulloblastoma, pancreatic cancer, breast cancer, ovarian cancer, small-cell lung cancer, and colorectal cancer 10-14. Singh et al. found that inhibition of ALK tyrosine kinase down-regulates SHH signaling pathway in ALK- positive anaplastic large cell lymphoma 14. Yoo et al. also found that SHH signaling pathway promotes motility and invasiveness of gastric cancer cells through TGF-beta-mediated activation of the ALK5-Smad 3 pathway 12. Park et al. found that cetuximab resistance in metastatic colorectal cancer is associated with SHH signaling pathway activation 15. Vismodegib and sonidegib are SHH signaling pathway inhibitor drugs via inhibiting SMO approved by the FDA. Recently, a randomized phase II trial shows that vismodegib does not increase the efficacy of standard therapy for metastatic colorectal cancer 16, as well as pancreatic cancer 17 and ovarian cancer 18. Therefore, a better understanding of the specific mechanism of SHH signaling pathway activation will help to facilitate anti-cancer drugs.

ALK has been described in a range of human cancers involved in cancer initiation and progression via activating multiple signaling pathways, such as the PI3K-AKT, CRKL-C3G, MEKK2/3-MEK5-ERK5, JAK-STAT and MAPK signaling pathways 19-23. Recently ALK and LTK ligand 1 (ALKAL1) also named “augmentor-β” or “FAM150A” was identified as a potent activating ligands for human ALK that bind to the extracellular domain of ALK 24, 25. However, due to its poor stability, the underlying mechanisms of ALKAL1 regulatingthe tumor progression in the human cancers including colorectal cancer have not been well documented.

In this study, we found that ALKAL1 was upregulated in colorectal cancer tissues and cell lines. Upregulation of ALKAL1 correlated with tumor malignancy and poor prognosis in colorectal cancer. Moreover, ALKAL1 silencing inhibited tumorigenesis, migration and invasion of colorectal cancer cells. Additionally, we found ALKAL1 silencing inhibited SHH signaling pathway, which is essential for ALKAL1 induced migration. In the current study, we give an update on what we know so far of ALKAL1 in colorectal cancer progression.

## Materials and methods

### Cell culture

The human colorectal cancer cell lines Caco-2, DLD-1, HCT-8, LS 174T, RKO, SW480, W620, T84 were obtained from the Shanghai Chinese Academy of Sciences Cell Bank (China). Colonic epithelial cells and rectal epithelial cells were isolated from healthy person of Jiangmen Central Hospital (Guangdong, China). All cells were maintained in DMEM basic medium (Gibco, Grand Island, NY, USA) with 1% penicillin G (100 U/ml), streptomycin (100 mg/ml) and 10% fetal bovine serum (Life Technologies) and were grown under a humidified atmosphere of 5% CO_2_ at 37 °C.

### Patients and tumor tissues

Paraffin-embedded 377 cases colorectal cancer tissues sections, 80 cases benign colorectal lesions, and 20 cases colorectal precancerous lesions were obtained during surgery, and the clinicopathological features of the patients are respectively summarized in Additional file 1: Table S1, Additional file 2: Table S2, Additional file 3: Table S3. 10 pairs of colorectal cancer tissues and adjacent normal tissues were collected immediately after surgery and stored at -80 °C. The clinicopathological features of these patients are summarized in Additional file 4: Table S4. All tissues were collected from the Affiliated Jiangmen Hospital of Sun Yat-sen University (Guangdong, China) between January 2010 and December 2018. Patients were diagnosed based on clinical and pathological evidence. For the use of these clinical materials for research purposes, prior patients' consents and approval from the Institutional Research Ethics Committee were obtained. The proportions of tumor vs. non-tumor in hematoxalin & eosin (HE) -stained tissue samples were evaluated by two independent professional pathologists.

### RNA extraction and quantitative PCR

Total RNA was extracted using RNAiso Trizol reagent (Takara, Kyoto, Japan), PrimeScript™ RT Master Mix (Takara, Japan) was used for reverse transcription according to the manufacturer's instructions. cDNA was amplified and quantified by Applied Biosystems 7500 (Thermo Fisher, USA) using SYBR Green (TaKaRa, Japan). GAPDH was used as internal controls, Primers were synthesized and purified by RiboBio (Guangzhou, China) and the primers are listed in Additional file 5: Table S5. The fold changes were determined using the relative quantification 2^-△△CT^ method.

### Western blot

Nuclear/cytoplasmic fractions were separated by the Cell Fractionation Kit (CST, USA) according to the manufacturer's instructions, and whole cell lysates were extracted using RIPA Buffer (CST, USA). Protein concentration was determined using a standard Bradford assay (Beyotime, China) and 50 μg per sample was separated by SDS-PAGE and transferred to a PVDF membrane (Millipore, USA) and detected using the following primary antibodies: ALKAL1 (Thermo Fisher, PA5-55591, 1:1000), α-tubulin (CST, 3873s, 1:2000), LIIMK1 (CST, 3842s, 1:1000), ROCK1 (CST, 4035s, 1:1000), MMP3 (CST, 143513s, 1:1000), MMP9 (CST, 13667s, 1:1000), GLI1 (CST, 3538s, 1:1000), PTCH (CST, 2468s, 1:1000) and p84 (Abcam, ab102684, 1:1000). Secondary antibodies conjugated to HRP were used prior to reading on the Azure Biosystems c400. α-tubulin served as the cytoplasm fractions and p84 severed as the nuclear marker.

### Immunohistochemistry (IHC)

Immunohistochemical staining was performed on 4 μm tissue sections using an EnVision™ Kit (DAKO, Denmark). After deparaffinized, rehydrated in graded ethanol, antigen retrieval and blocking, the slides were incubated overnight at 4 °C in a humidified chamber with anti-ALKAL1 antibodies (Thermo Fisher, PA5-55591), Vimentin (sc-373717), Ki67 (27309-1-AP, Proteintech) and caspase 3 cleavage (Asp175, 9661, Cell Signaling Technology) diluted 1:50 in PBS. Scores given by two independent Pathologists were averaged for further comparative evaluation of ALKAL1 expression. The details of scoring criteria were described in our previous study 26, 27. According to this method, ALKAL1 expression in colorectal cancer samples was evaluated by the staining index, with scores of 0, 1, 2, 3, 4, 6, 8, 9 or 12. High or low expression of ALKAL1was stratified according to the following criteria: SI ≤ 4 was defined as tumors with low expression of ALKAL1, and SI ≥ 6 was defined as tumors with high expression of ALKAL1.

### Vectors and transient transfection

Plasmid construction and transfection Sh-ALKAL1 with control vector were designed by GenePharma (Suzhou, China). The short hairpin (shRNA) RNA for human ALKAL1 was cloned into a hU6-MCS-CBh-gcGFP-IRES-puromycin lentiviral vector (GV493, Genechem, China), and the list of primers used in clone reactions was presented in Additional file 6: Table S6. Colorectal cancer cells were cultured in 6-well plates to reach 60~70% confluence, then transfected using Lipofectamine 3000 (Thermo Fisher, USA) according to the manufacturer's protocol.

### Cell counting kit-8 analysis and colony formation assay

Cell proliferation was measured using a Cell Counting Kit-8 (CCK-8, Dojindo, Kumamoto, Japan), 1 × 10^3^ colorectal cancer cells were seeded into 96-well plate for 1 d~5 d, then cells were incubated with 10 μL CCK-8 for 4 hours. Absorbance of cells was measured at 450 nm. For colony formation assay, 0.5 × 10^3^ cells were plated into six-well plate and cultured in a humidified atmosphere containing 5% CO_2_ at 37 °C for 10 days. Colonies were then fixed for 30 min with 10% formaldehyde and stained for 30 min with 1.0% crystal violet. Colony morphologies were captured under a light microscope (Olympus).

### Cell cycle analysis

1×10^6^ colorectal cancer cells were harvested by trypsinization, washed with cold phosphate-buffered saline (PBS) twice and fixed in 75% ice-cold ethanol in PBS overnight. Before staining, cells were gently resuspended in cold PBS, and ribonuclease was added to corresponding tube, and incubated at 37 °C for 30 min, followed by incubation with propidium iodide (Dojindo, Japan) for 20 min at room temperature. Cell samples were then analyzed by BD FACS Calibur II (San Jose, CA, US) and analyzed using FlowJo 7.6 software (TreeStar Inc., Ashland, OR, USA).

### Anchorage-independent growth ability assay

Cells (3×10^3^) were suspended in 2 ml complete medium plus 0.3% agar (Sigma-Aldrich, St Louis, MO, US). The agar-cell mixture was plated as a top layer onto a bottom layer of 0.6% complete medium agar mixture. After 14 days, the colony size was measured through an ocular micrometer and colonies in diameter > 0.1 mm were counted.

### Animal experiments

Animal handling and experimental procedures were approved by the Animal Experimental Ethics Committee of Guangdong Medical University. 4-5 week old female BALB/c nude mice were purchased from the Shanghai Experimental Animal Center of the Chinese Academy of Sciences. 6 nude mice per group were used to ensure the adequate power and each mouse with different weight was randomly allocated, SW480 cells transfected with sh-ALKAL1 or vector diluted to a concentration of 1×10^7^ cells/ml in cold PBS. A total of 200 μL of suspended cells was subcutaneously injected into the left flank area of each mouse. Mouse body weight was examined 3 times per week, and tumor size was calculated with calipers (0.5 × length × width^2^). After 5 weeks, the mice were sacrificed and individual tumors were harvested and weighed, Sections (4 μm) of tumors were mounted on glass slides and stained with HE.

### Wound-healing assay

Cell migration was assessed using a wound-healing assay. *ALKAL1* knockdown or shRNA control cells were cultured to ~100% confluence in 24 well plate, a wound was generated by scratching the monolayer with the tip of a 10 µl pipette, then removed old medium and washed three times with PBS to remove cell fragments and continuously cultured. The wound was photographed at 0 h, 24 h and 48 h using a microscope (Olympus, Tokyo, Japan). The relative distance between the gaps was photographed and measured under an inverted microscope (Olympus).

### Migration and invasion assays

For the migration assay, the cells were suspended at a density of 2×10^5^ cells/ml. For the invasion assay, the upper chamber was pre-coated with diluted Matrigel (BD, San Jose, CA, USA). Matrigel was coagulated after 3 h, and the cells were suspended in DMEM at a density of 5×10^5^ cells/ml, 100 μl cell suspension was then seeded into the upper chamber (Corning, 8 μm). To attract the cells, 600 μl DMEM containing 20% FBS was added in the lower chamber. After incubation (16 h for invasion and 24 h for migration), the cells that migrated through the membrane were fixed for 30 min with methanol and stained for 30 min with 0.05% crystal violet (Beyotime) at room temperature. The number of migrated or invasive cells was counted from five random fields under an optical microscope (Olympus).

### Dual luciferase report experiment

The dual luciferase report assay was performed according to a published method with minor modifications 2, 26. Briefly, 5×10^5^ cells were plated in 6 cm cell culture dishes, then transfected with reporter constructs or the negative control using Lipofectamine 3000. Luciferase and Renilla luciferase were measured using a Dual-Luciferase Reporter Assay System (Promega) according to the manufacturer's instructions. The luciferase activity of each lysate was normalized to the Renilla luciferase activity. The relative transcriptional activity was converted to the fold induction above the vehicle control value.

### Statistical analysis

All values are presented as the mean ± standard deviation (SD). Significant differences were determined using GraphPad Prism 5.0 software (USA). Student's t-test was used to determine significant differences between two groups. Between-group comparisons were conducted using single-factor analysis of variance (one-way ANOVA). The chi-square test was used to analyze the relationship between ALKAL1 expression and clinicopathological characteristics. Survival analysis was evaluated using the Kaplan-Meier method and assessed using the log-rank test. *P* < 0.05 was statistically significant.

## Results

### ALKAL1 is upregulated in colorectal cancer tissues and cell lines

**Figure 1:**
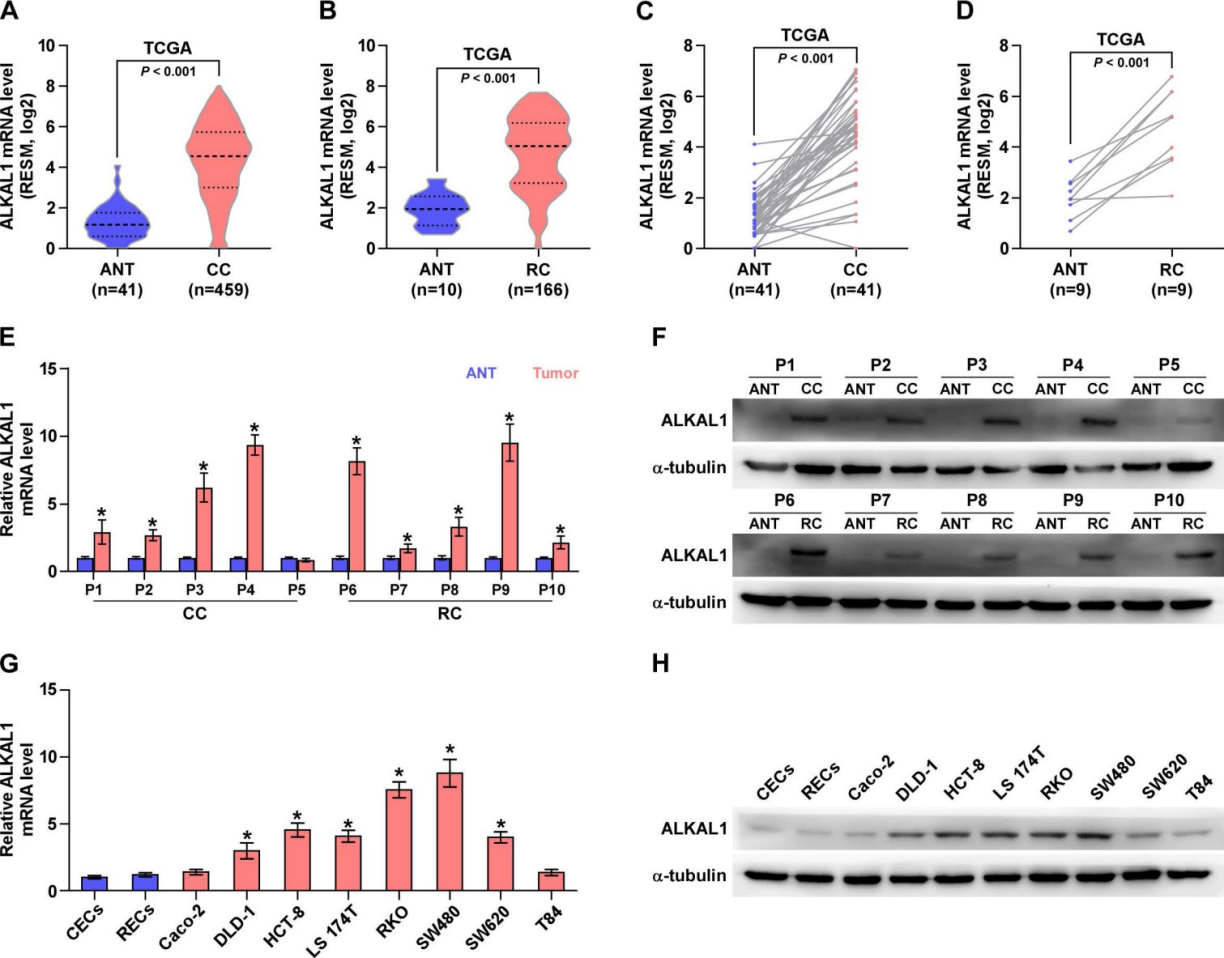
** ALKAL1 is upregulated in colorectal cancer tissues and cell lines. A-D)** ALKAL1 expression levels was markedly upregulated in colorectal cancer tissues as assessed by analyzing TCGA colorectal cancer miRNA sequencing datasets. **(E)** Real-time PCR analysis of *ALKAL1* in 10 primary colorectal cancer tissues compared with the matched adjacent normal tissues (ANT). **(F)** ALKAL1 protein expression in 10 paired CRC and adjacent normal tissues (ANT) from the same patient were detected by Western blot. α-tubulin was used as the loading control. **(G)** *ALKAL1* mRNA expression was determined by RT-PCR in selected 8 colorectal cancer cell lines (Caco-2, DLD-1, HCT-8, LS 174T, RKO, SW480, SW620 and T84). **(H)** ALKAL1 protein expression was determined by Western blot in selected 8 colorectal cancer cell lines (Caco-2, DLD-1, HCT-8, LS 174T, RKO, SW480, SW620 and T84).

Aberrant activation of ALK has been described in a range of human cancers, including anaplastic large cell lymphoma, non-small cell lung cancer, neuroblastoma, inflammatory myofibroblastic tumors, colorectal cancer, and so on 28. ALKAL1 is an activating ligands for ALK 24, 25. Herein, we analyzed colorectal cancer RNA sequencing datasets from The Cancer Genome Atlas (TCGA) and found that ALKAL1 expression was upregulated in colorectal cancer tissues compared with adjacent normal tissues (Figure 1A-D). Furthermore, we assessed whether ALKAL1 expression was elevated in our own 10 paired CRC tissues. Consistent with TCGA analysis, we found that ALKAL1 was upregulated in CRC tissues and high expression of ALKAL1 was seen in the 9/10 primary CRC tissue samples compared with the matched adjacent normal tissue samples (Figure 1E, 1F). In order to further confirm whether ALKAL1 is expressed in colorectal cancer cells, *ALKAL1* mRNA and protein expression were examined in selected 8 colorectal cancer cell lines (Caco-2, DLD-1, HCT-8, LS 174T, RKO, SW480, SW620 and T84), and the results showed that ALKAL1 is detectable in colorectal cancer epithelial cell lines (Figure 1G, 1H). Especially ALKAL1 showed high level expression in DLD-1, HCT-8, LS 174T, RKO, SW480 and SW620 cell lines, when compared with colorectal normal cell lines (CECs and RECs) or colorectal cancer Caco-2 or T84 cell lines (Figure 1G, 1H). Notably, RKO and SW480 cells that highly expressed ALKAL1 are cell lines derived from patients with poorly differentiated colorectal cancer. These studies indicate that the high expression of ALKAL1 in patients with colorectal cancer may be related to the malignancy of patients with colorectal cancer.

### Upregulation of ALKAL1 correlates with tumor malignancy and poor prognosis in colorectal cancer

**Figure 2:**
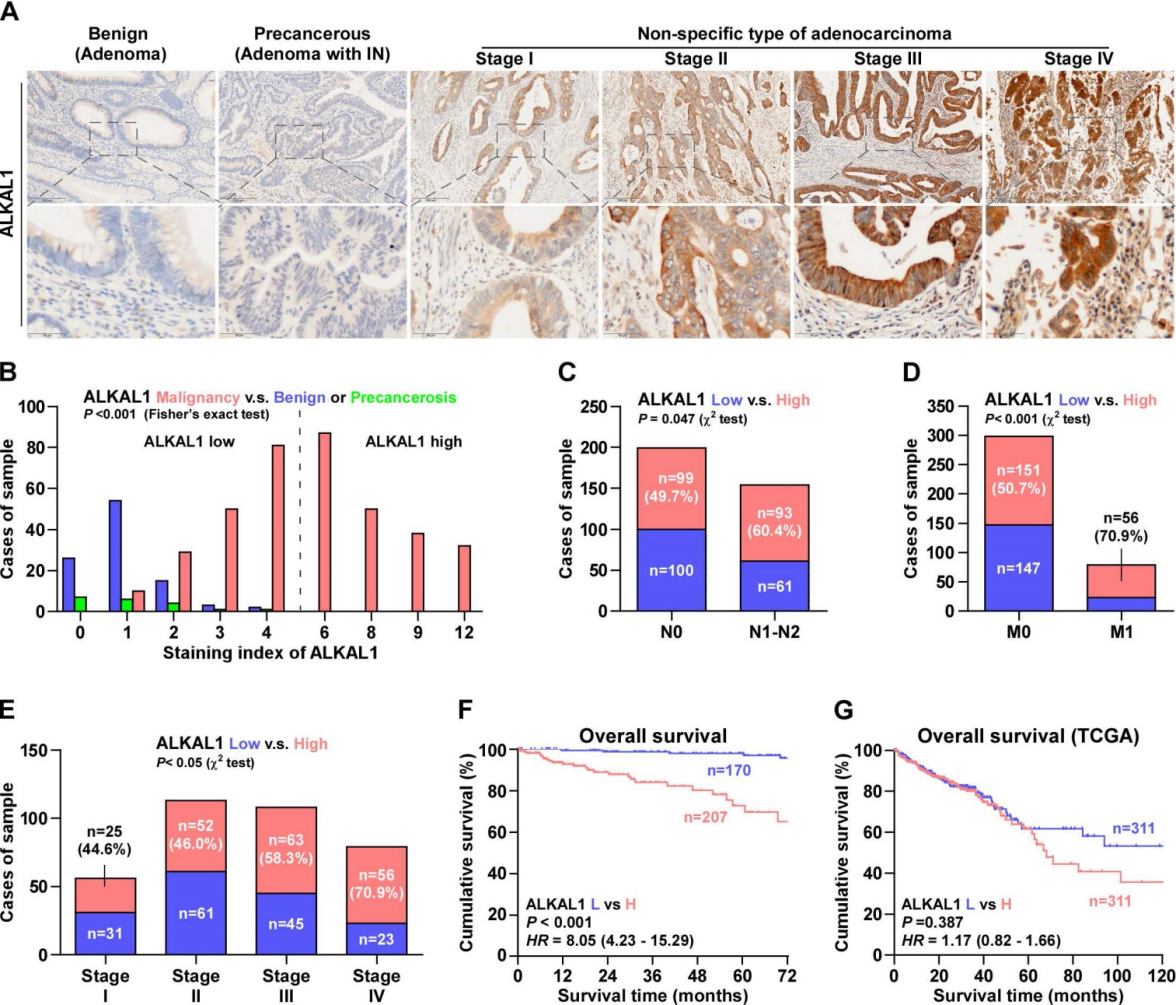
** Upregulation of ALKAL1 correlates with tumor malignancy and poor prognosis in colorectal cancer. (A)** Representative photomicrographs of immunohistochemical analysis for ALKAL1 expression at various pathological stages, including benign, precancerous, and tumor stages I to IV. ALKAL1 was stained in brown. **(B)** Distribution of ALKAL1 immunostaining index across a variety of pathological stages of colorectal cancer. High expression of ALKAL1 was observed in 240/353 colorectal cancer tissue samples. **(C-E)** High expression of ALKAL1 was strong positive correlated with tumor classification, node classification and pathological stages. **(F)** Kaplan-Meier survival analysis revealed that patients with high ALKAL1 expression had poor overall survival. **(G)** Kaplan-Meier analysis of overall survival curves of colorectal cancer patients datasets from TCGA.

To further reveal the relationship between ALKAL1 and tumor malignancy in colorectal cancer. 377 cases human colorectal cancer tissue samples were used to detect ALKAL1 expression by immunohistochemical analysis. The results showed that ALKAL1 expression was primarily detected within the cytoplasm and the ALKAL1 expression positively correlated with clinical stages (Figure 2A). In addition, high expression of ALKAL1 was observed in 207/377 colorectal cancer tissue samples (Figure 2B). High expression of ALKAL1 was strong positive correlated with tumor classification, node classification and pathological stages (Figure 2C-2E, Table S7). Kaplan-Meier survival analysis revealed that patients with high ALKAL1 expression had poor overall survival (Figure 2F), which were consistent with TCGA (Figure 2G). These studies suggest that upregulation of ALKAL1 correlates with tumor malignancy and poor prognosis in colorectal cancer.

### ALKAL1 silencing is not associated with colorectal cancer cell proliferation

**Figure 3:**
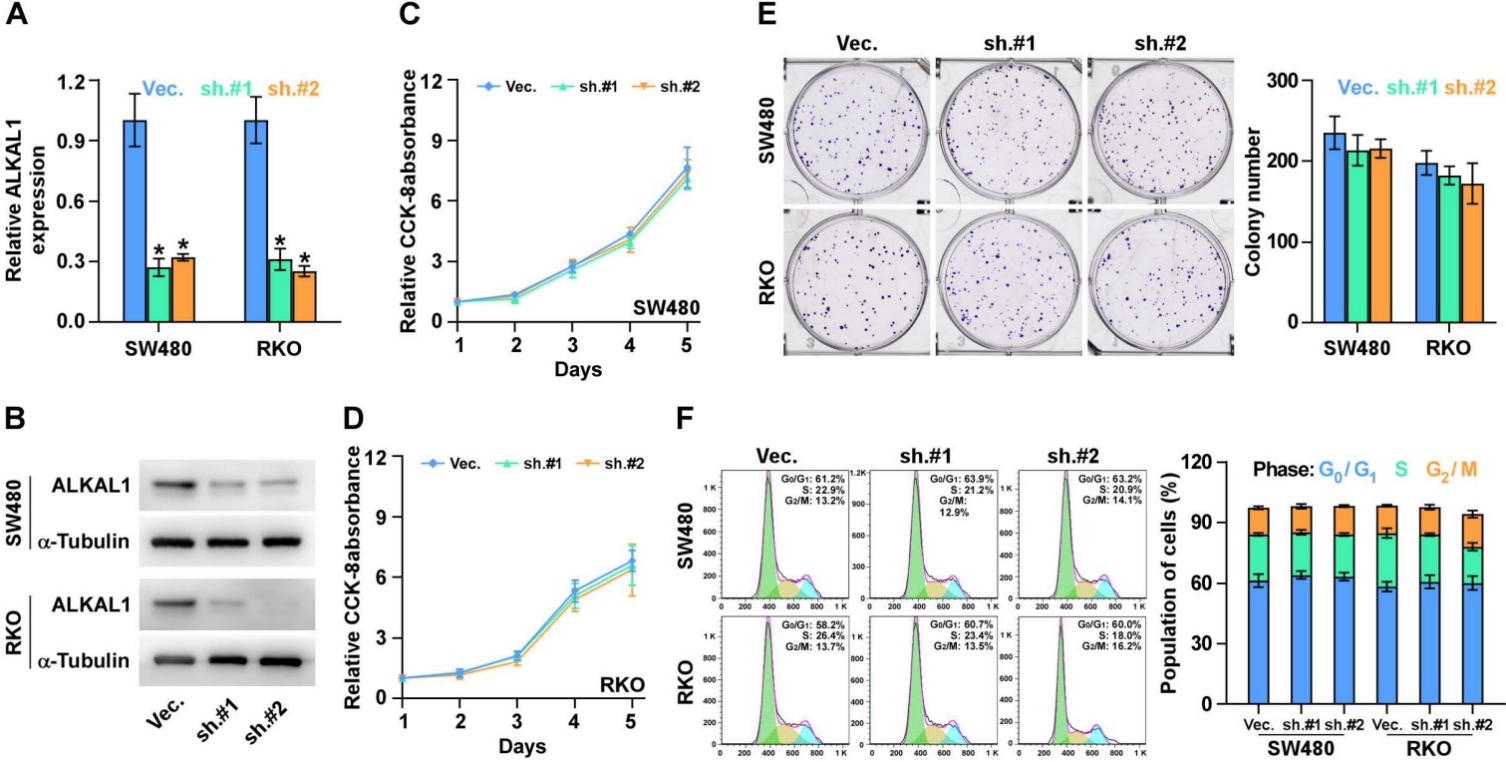
** ALKAL1 silencing is not associated with colorectal cancer cell proliferation. (A)** Real-time PCR analysis of *ALKAL1* expression in ALKAL1 silencing RKO and SW480 cell lines by endogenously knocking down ALKAL1 with retrovirus (sh.#1 and sh.#2) infection. **(B)** Western blot analysis of ALKAL1 expression in ALKAL1 silencing RKO and SW480 cell lines by endogenously knocking down ALKAL1 with retrovirus (sh.#1 and sh.#2) infection. **(C-D)** CCK-8 analysis of RKO and SW480 cells proliferation. **(E)** Plate colony formation analysis of RKO and SW480 cell by endogenously knocking down ALKAL1 with retrovirus (sh.#1 and sh.#2) infection. **(F)** Cell cycle analysis with PI staining analysis of RKO and SW480 cells by endogenously knocking down ALKAL1 with retrovirus (sh.#1 and sh.#2) infection. Error bars represent the mean ± S.D. of three independent experiments. *P < 0.05.

To determine the biological roles of ALKAL1 in colorectal cancer, we constructed ALKAL1 silencing RKO and SW480 cell lines by endogenously knocking down ALKAL1 with retrovirus (sh.#1 and sh.#2) infection (Figure 3A, 3B). Real-time PCR and western blot were performed to identify the mRNA and protein levels of ALKAL1 expression. It is worth noting that ALKAL1 silencing is not associated with colorectal cancer RKO and SW480 cell proliferation (Figure 3C, 3D), plate colony formation (Figure 3E) and cell cycle distribution (Figure 3F) via CCK-8 assay, plate clone formation assay and cell cycle analysis with PI staining using Flow cytometry, respectively.

### ALKAL1 silencing inhibits colorectal cancer cell tumorigenesis

**Figure 4:**
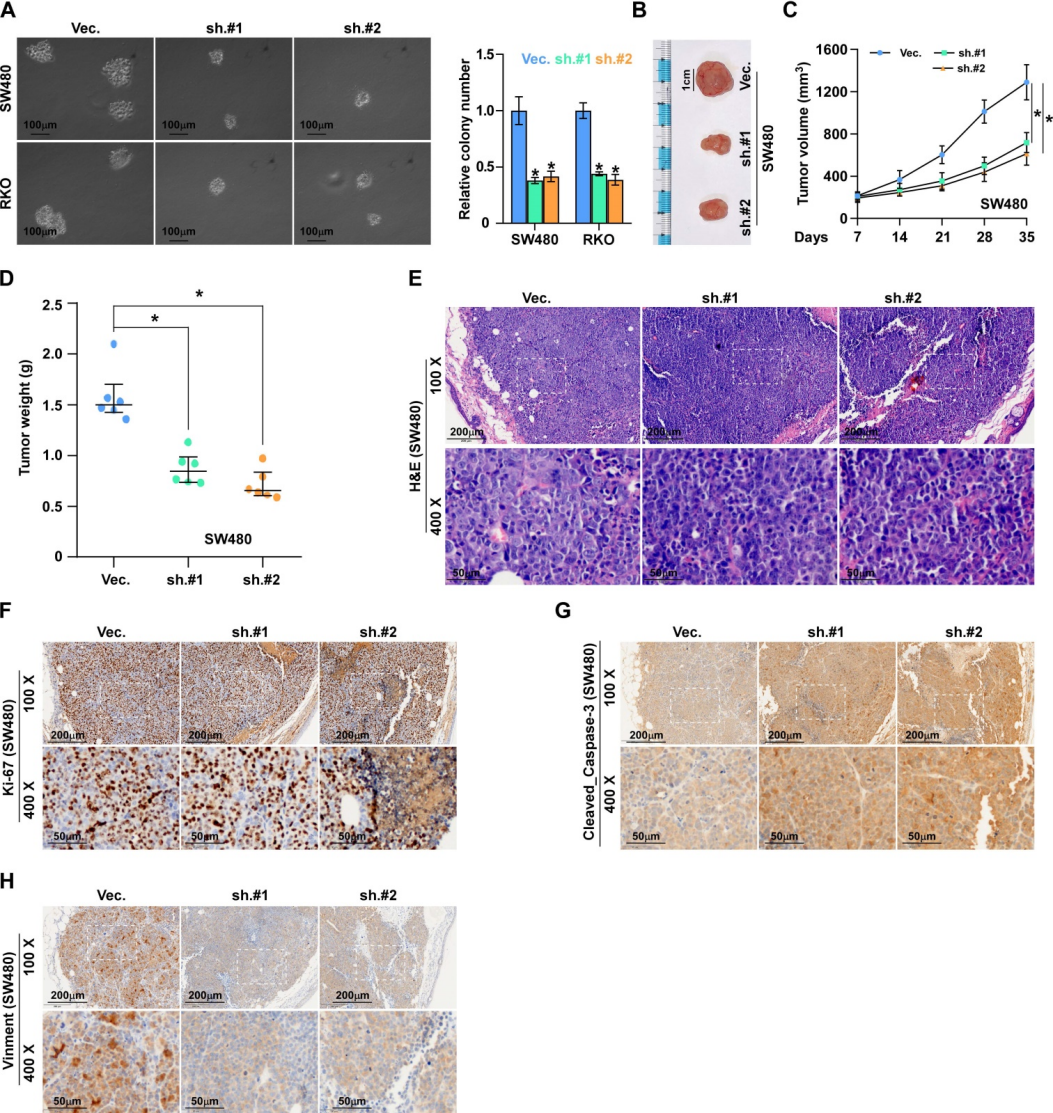
** ALKAL1 silencing inhibits colorectal cancer cell tumorigenesis. (A)** Anchorage-independent growth assay analysis of the anchorage-independent growth ability of ALKAL1-silenced colorectal cancer cells. **(B)** Representative images of excised tumors after injection of SW620 cells transduced with ALKAL1-silencing shRNA or vector control. **(C)** Tumor volumes were measured every week after the seventh day post injection (n = 6, *p* < 0.05). **(D)** Tumor weights were determined after tumor-bearing mice were sacrificed (n = 6, *p* < 0.05). **(E)** HE staining analysis of tumor from tumor-bearing mice. **(F-H)** Representative images of Ki67, vimentin and caspase 3 cleavage immunostaining in xenograft tumors that transduced with ALKAL1-silencing shRNA or vector control. Magnification: 100× for the upper panel, and 400× for the lower panel.

To investigate the effects of ALKAL1 on the tumorigenic activity of colorectal cancer cells, we performed an anchorage-independent growth assay and found that ALKAL1 silencing reduced the anchorage-independent growth ability of colorectal cancer cells (Figure 4A). We next evaluated the effect of ALKAL1 on tumorigenesis *in vivo*. As a result, we found that the tumors formed by the ALKAL1-silenced cells were smaller, and the tumor volumes and weight were decreased significantly in the ALKAL1 silencing group compared with the control group (Figure 4B-D). The HE staining results showed that the ability of ALKAL1-silenced cancer cells in the tumor of mice to invade surrounding cells was significantly reduced (Figure 4E). Additionally, the expression of vimentin, Ki67 and caspase 3 cleavage in the tumors were detected by IHC (Figure 4F-H). Results showed that ALKAL1-silenced tumors, the vimentin and caspase 3 expression were down-regulated (Figure 4G-H). However, there is no difference in the ki-67 expression after ALKAL1 silencing (Figure 4F).

### ALKAL1 silencing inhibits migration and invasion of colorectal cancer cells

**Figure 5:**
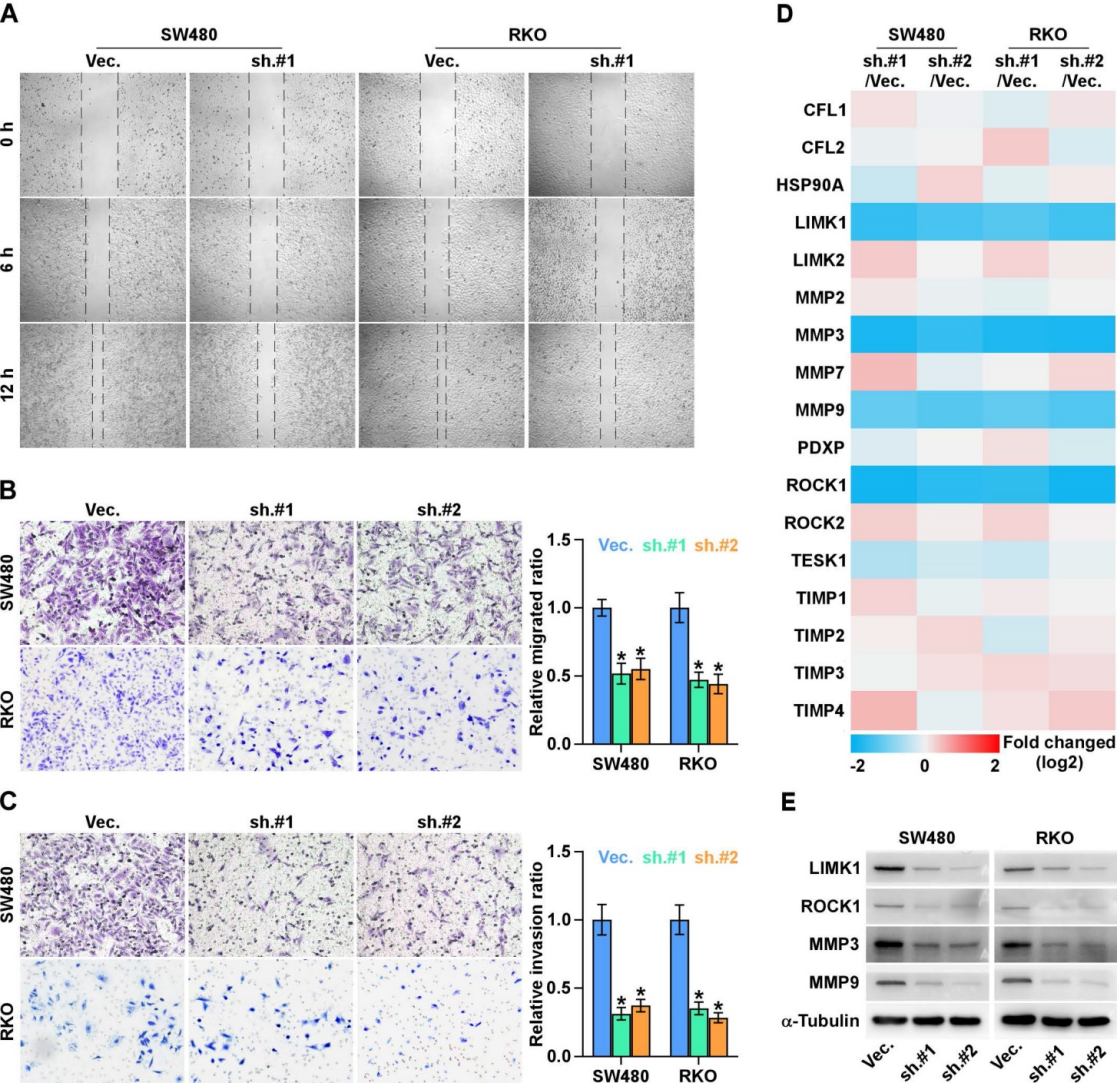
** ALKAL1 silencing inhibits metastasis and invasion of colorectal cancer cells. (A)** Wound healing and migration assay analysis of the migration ability of ALKAL1 silencing colorectal cancer cells. **(B-C)** Transwell cell invasion and migration assay analysis of the invasion and migration ability of ALKAL1 silencing colorectal cancer cells. **(D)** Real-time PCR analysis of some selected gene (*ROCK1, ROCK2, TESK1, PDXP, LIMK1, LIMK2, HSP90AA1, CFL1, CFL2, MMP2, MMP3, MMP7, MMP9, TIMP1, TIMP2, TIMP3* and *TIMP4* ) expression that related to cell movement and invasion in ALKAL1 silencing RKO and SW480 cell lines. **(E)** Western blot analysis of ROCK1, LIMK1, MMP3, and MMP9 expression in ALKAL1 silencing RKO and SW480 cell lines.

To investigate the effects of ALKAL1 on the metastatic ability of colorectal cancer cells, we performed cell wound healing and migration assay and found ALKAL1 silencing reduced the migration ability of colorectal cancer cells (Figure 5A). Similarly, transwell cell invasion and migration assay also showed that downregulating ALKAL1 decreased the invasion and migration ability of colorectal cancer cells (Figure 5B, 5C). In addition, we detected mRNA expression levels in some selected gene (*ROCK1, ROCK2, TESK1, PDXP, LIMK1, LIMK2, HSP90AA1, CFL1, CFL2, MMP2, MMP3, MMP7, MMP9, TIMP1, TIMP2, TIMP3* and *TIMP4* ) that related to cell movement and invasion (Figure 5D). Results showed that ROCK1, LIMK1, MMP3, and MMP9 mRNA expressions were down-regulated after ALKAL1 silencing in colorectal cancer RKO and SW480 cells. Consistently, western blot results also showed that silencing ALKAL1 could inhibit ROCK1, LIMK1, MMP3, and MMP9 protein expression (Figure 5E). These studies suggest that ALKAL1 silencing inhibits migration and invasion of colorectal cancer cells.

### ALKAL1 silencing inhibits SHH signaling pathway

**Figure 6:**
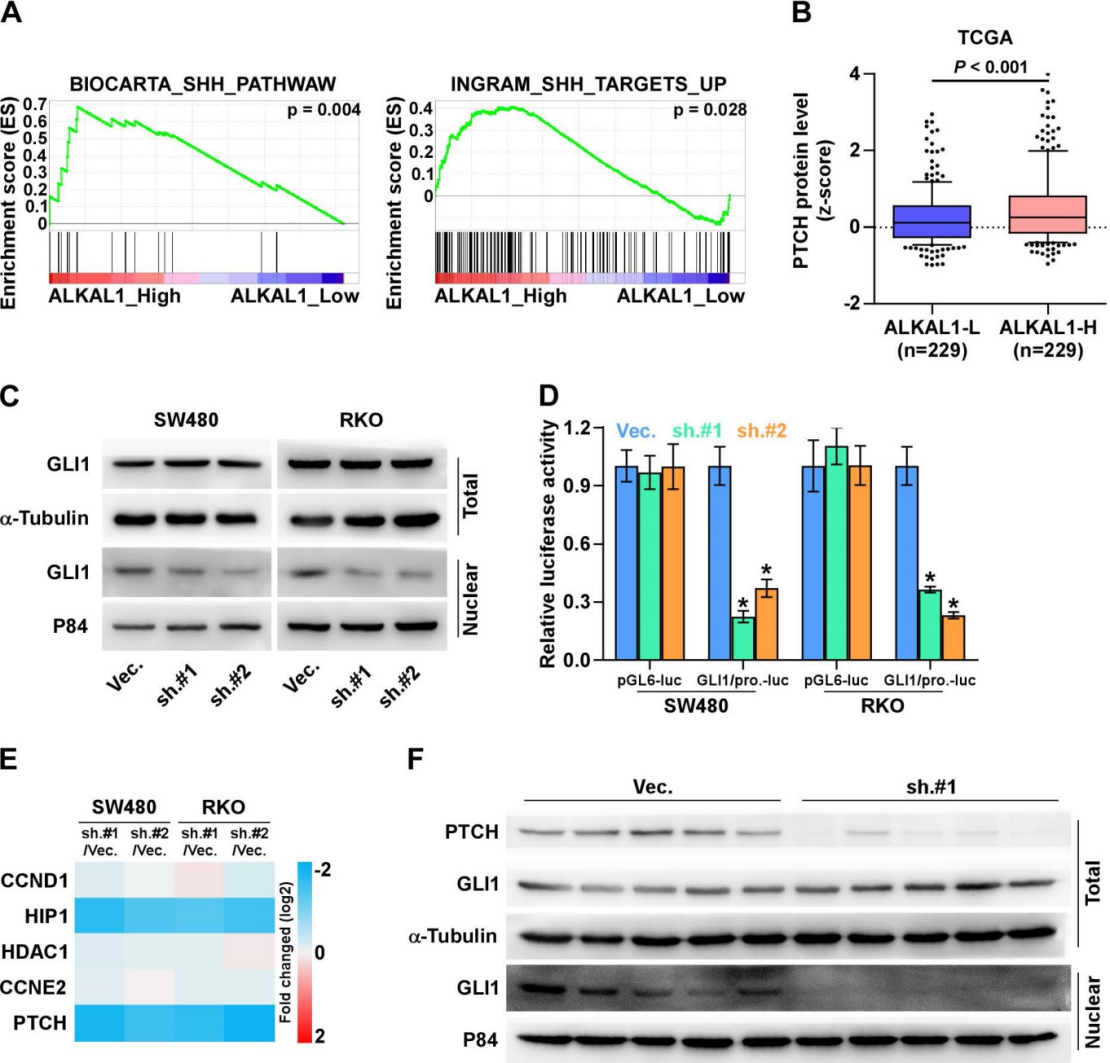
** (A) ALKAL1 silencing inhibits SHH signaling pathway.** A gene set enrichment analysis (GSEA) of ALAK1 expression against SHH signaling pathway. **(B)** ALKAL1 high expression significantly and positively correlated with PTCH protein levels from TCGA. **(C)** Western blot analysis of nuclear and total level of GLI1 in ALKAL1 silencing RKO and SW480 cell lines. **(D-E)** Luciferase assay analysis of GLI1 dependent luciferase activity and the expression levels of multiple downstream genes in ALKAL1 silencing RKO and SW480 cells. **(F)** Western blot analysis of PTCH and GLI1 in subcutaneous xenografts of nude mice.

The Sonic hedgehog (SHH) signaling pathway is closely linked to tissue polarity, patterning and stem cell renewal during embryonic development. Previous studies have demonstrated that the SHH signaling pathway also plays an important role in proliferation, angiogenesis, stemness, and metastasis in various cancers including colorectal cancer. We further examined the role of ALKAL1 in SHH signaling pathway in colorectal cancer cells. As shown in Figure 6A, we performed a gene set enrichment analysis (GSEA) of ALAK1 expression against SHH signaling pathway, and found ALKAL1 high expression significantly and positively correlated with SHH signatures (“BIOCARTA_SHH_PATHWAW” and “INGRAM_SHH_TARGETS_UP”). In vertebrates, the binding of Hedgehog ligands (Sonic, Indian, and Desert Hedgehog) to their receptor, patched (PTCH), results in activation of the pathway. Herein, we analyzed colorectal cancer RNA sequencing datasets from TCGA and found that ALKAL1 high expression also significantly and positively correlated with PTCH protein levels (Figure 6B). GLI1 is the major transcriptional activator of the Hedgehog target genes. Western blot results also showed that ALKAL1 silencing inhibited nuclear level of GLI1, but does not affect total protein levels of GLI1 (Figure 6C). Furthermore, we found that ALKAL1 silencing significantly decreased GLI1 dependent luciferase activity as well as the expression levels of multiple downstream genes, including PTCH, HIP1 but not CCND1, CCNE2 and HDAC1 in RKO and SW480 cells (Figure 6D, 6E). Moreover, western blot analysis of PTCH and GLI1 in subcutaneous xenografts of nude mice showed that PTCH and nuclear GLI1 were downregulated in tumors formed by the ALKAL1-silenced cells (Figure 6F). These results suggest that ALKAL1 may regulate the SHH signaling pathway.

### SHH signaling pathway activation is essential for ALKAL1 induced migration

**Figure 7:**
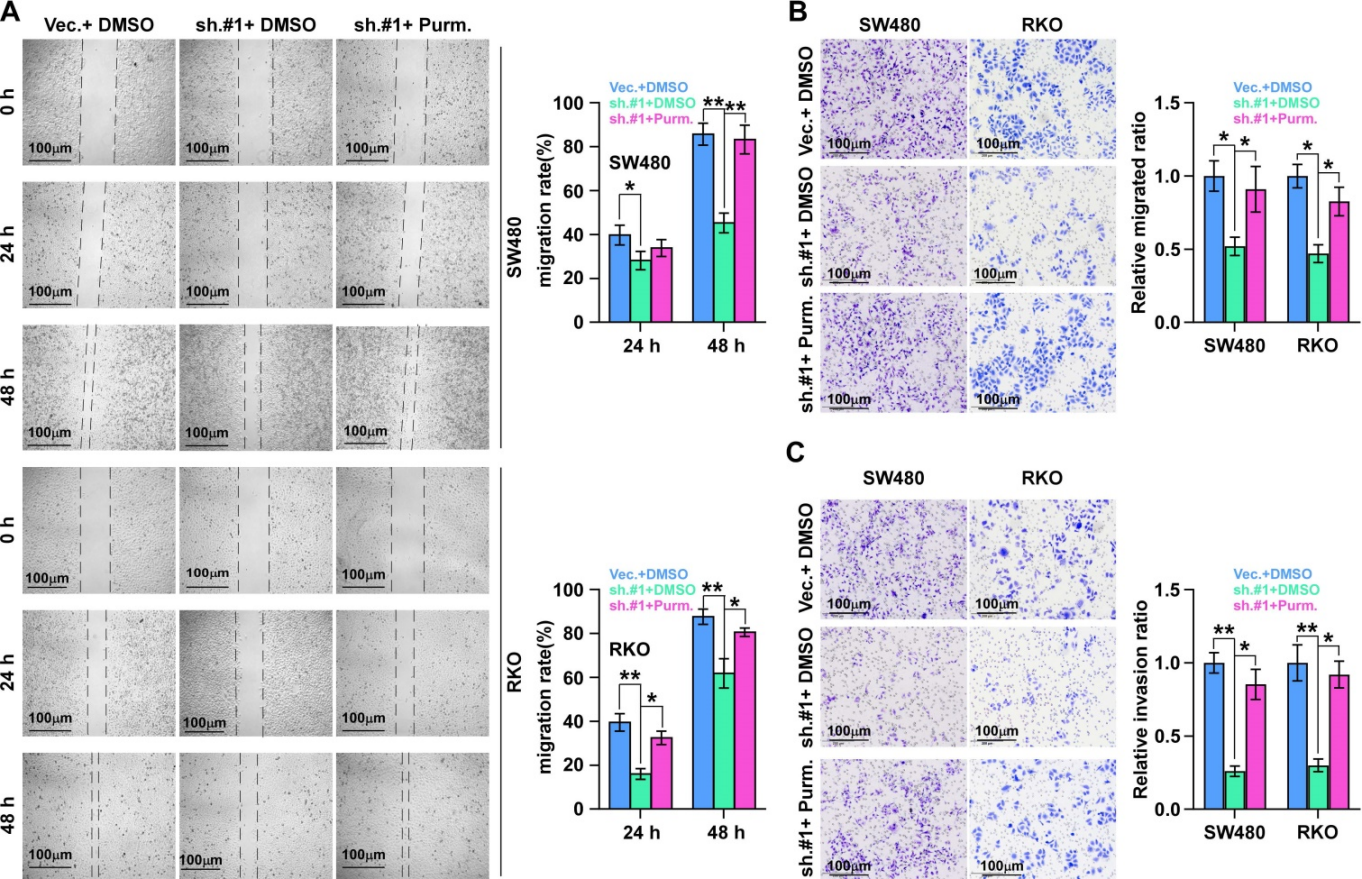
** SHH signaling pathway activation is essential for ALKAL1 induced migration. (A)** Wound healing and migration assay analysis of the role of SHH signaling pathway activation in ALKAL1 induced migration using SHH signaling pathway agonist purmorphamine (Purm). **(B-C)** Transwell cell invasion and migration assay analysis of the invasion and migration ability in ALKAL1 induced migration using SHH signaling pathway agonist purmorphamine (Purm).

We then detected the role of SHH signaling pathway activation in ALKAL1 induced migration using SHH signaling pathway agonist purmorphamine by performing cell wound healing and migration assay and transwell cell invasion and migration assay. As shown in Figure 7, activation of SHH signaling pathway with purmorphamine (2.5 μmol/L) promoted the invasion and migration ability of ALKAL1-silenced RKO and SW480 cells (Figure 7A-C). These results suggest that SHH signaling pathway activation is essential for ALKAL1 induced migration and invasion.

## Discussion

Recently, anaplastic lymphoma kinase (ALK) and leukocyte tyrosine kinase (LTK) have been identified as “orphan” receptor tyrosine kinases (RTKs) with carcinogenic potential. Wild-type ALK is a membrane-bound receptor. In general, ALK activates multiple signaling pathways, such as the PI3K-AKT, CRKL-C3G, MEKK2/3-MEK5-ERK5, JAK-STAT and MAPK signaling pathways 19, which involved in the initiation and progression of many different cancer types, including lymphomas, neuroblastoma and colorectal cancer. Bavi et al. reported that ALK gene amplification was associated with poor prognosis in colorectal cancer using fluorescence *in situ* hybridisation (FISH) and immunohistochemistry as a screening tool to detect the prevalence of ALK copy number changes, translocations, gene mutations and protein expression 21. Pietrantonio et al. found ALK, ROS1, and NTRK rearrangements can define a new rare subtype of metastatic colorectal cancer with extremely poor prognosis 20. Recently, Nouri et al. also identifies ALK as a novel regulator of the Hippo pathway in tumorigenesis and immune evasion via a kinome-wide screen using a NanoLuc LATS luminescent biosensor 29. LTK is a tyrosine kinase that has been suggested to be specific for hematopoietic cells and neuronal cells and reported as an unusual membrane protein lacking an extra cellular domain. The LTK pathway has been implicated in autoimmunity, neuronal development, and cancer 30. Muller-Tidow et al. found that LTK expression was increased in acute myeloid leukemia patients via a high-throughput kinase expression study 31. ALKAL1 was discovered as physiological ligands of ALK and LTK 23-25, 32. However, the role and molecular mechanisms of ALKAL1 in colorectal cancer are still poorly understood. In this study, we found that ALKAL1 was upregulated in colorectal cancer tissues and cell lines. Upregulation of ALKAL1 correlated with tumor malignancy and poor prognosis in colorectal cancer. Moreover, ALKAL1 silencing inhibited tumorigenesis, migration and invasion of colorectal cancer cells. Additionally, we found ALKAL1 silencing inhibited SHH signaling pathway, which is essential for ALKAL1 induced migration. Taken together, our results uncover a novel mechanism of ALKAL1 contributing to the activation of SHH signaling pathway in colorectal cancer progression.

A number of studies indicated that several mechanisms have been reported to be implicated in the constitutive activation of SHH signaling pathway in tumorigenesis. Activation of SHH signaling pathway can happen in canonical signaling pathway (by ligand-dependent interaction or through receptor-induced signaling) and non-canonical signaling pathway (activation downstream of smoothened [SMO]) 33. The canonical SHH signaling pathway occurs when the SHH binds and inactivates the 12-transmembrane protein Patched (PTCH). This downstream signaling cascade results in the translocation of GLI family proteins to the nucleus that begins the transcription of target genes, including PTCH1 and GLI1, in a negative and positive feedback loop, respectively. We focus here on colorectal cancer. We performed a gene set enrichment analysis (GSEA) of ALAK1 expression against SHH signaling pathway, and found ALKAL1 high expression significantly and positively correlated with SHH signatures (“BIOCARTA_SHH_PATHWAW” and “INGRAM_SHH_TARGETS_UP”). PTCH1 and GLI1 mRNA expression as an indication of the canonical SHH signaling pathway activity in tumor malignancy. Furthermore, we found that ALKAL1 silencing inhibited nuclear level of GLI1, and significantly decreased GLI1 dependent luciferase activity as well as the expression levels of multiple downstream genes, including PTCH, HIP1 but not CCND1, CCNE2 and HDAC1 in RKO and SW480 cells. These results indicated that ALKAL1 may regulate the canonical SHH signaling pathway.

**Figure 8:**
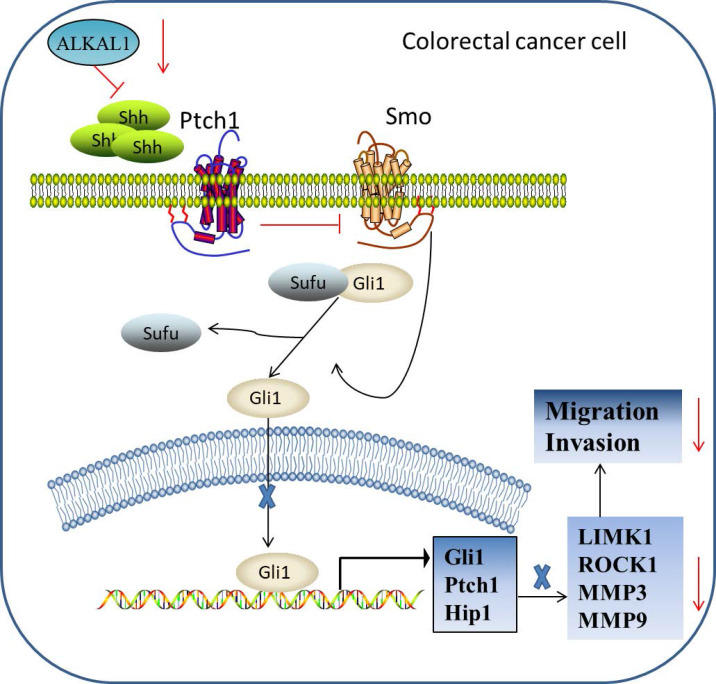
Hypothetical model illustrating that ALKAL1 plays an important role in colorectal cancer migration and invasion via activating SHH signaling pathway.

The report of biological role and clinical significance of ALKAL1 has been limited by difficulties in producing sufficient amounts of the ligands and its poor stability. Recently, Reshetnyak et al group reported that ALKAL1 was essential for embryonic iridophore development and adult body coloration in Zebrafish 23. Subsequently, Reshetnyak et al. further found that deletion of the N-terminal variable region minimally affects the activity of ALKAL1 toward LTK or ALK stimulation in cultured cells 22. So far, ALKAL1 has been rarely reported in cancer. In here, we give an update on what we know so far of ALKAL1 in colorectal cancer progression. Our results found that ALKAL1 was dramatically elevated in colorectal cancer tissues compared to the adjacent normal tissues and high expression of ALKAL1 correlated with poor prognosis in colorectal cancer patients. In ALKAL1 silenced tumors, the expression of vimentin and caspase 3 was down-regulated. However, there was no difference in the expression of ki-67 after ALKAL1 silence. Furthermore, ALKAL1 silencing inhibits migration and invasion, and SHH signaling pathway of colorectal cancer cells. However, the specific mechanism responsible for the improved outcomes in colorectal cancer patients with high ALKAL1 expression, the biological role of ALKAL1 overexpressing in colorectal cancer cell lines and the targets of ALKAL1 in SHH signaling pathway were not mentioned in this manuscript. In summary, our findings reveal that ALKAL1 plays an important role in colorectal cancer migration and invasion via activating SHH signaling pathway (Figure 8). Therefore, improved understanding of the specific role of ALKAL1 in the pathogenesis of colorectal cancer facilitates to increase our knowledge in colorectal cancer development.

## Supplementary Material

Supplementary tables.
