# Supplementary material for: ALKAL1 gene silencing prevents colorectal cancer progression via suppressing Sonic Hedgehog (SHH) signaling pathway
Source: J Cancer. 2021 Jan 1;12(1):150–62. doi: 10.7150/jca.46447 (PMC7738833; doi:10.7150/jca.46447)
Supplement: Supplementary file 1 — Supplementary tables. [file jcav12p0150s1.pdf]

Table S1. The basic information of 377 patients with colorectal cancer for ALKAL1 immunohistochemical staining analysis.

		Cases (n)	Percentage (%)
Location	Rectum	168	44.6
	Colon	196	52.0
	NA	13	3.4
Gender	Female	158	41.9
	Male	218	57.8
	NA	1	0.3
Age	<60	187	49.6
	⩾60	190	50.4
Histologic type	Non-specific[a]	316	83.8
	Other[b]	61	16.2
Grade	G1	28	7.4
	G2	253	67.1
	G3	27	7.2
	NA	69	18.3
T classification	T1	16	4.2
	T2	68	18.0
	T3	255	67.6
	T4	11	2.9
	NA	27	7.3
N classification	N0	199	52.8
	N1	91	24.1
	N2	63	16.7
	NA	24	6.4
M classification	M0	298	79.0
	M1	79	21.0
Stage	Stage I	56	14.9

Stage II	113	30.0
Stage III	108	28.6
Stage IV	79	21.0
NA	21	5.5

Noted: a) Non-specific type of adenocarcinoma included polypoid adenocarcinoma, tubular adenocarcinoma, and mucious adenocarcinoma. b) Other type included signet ring cell carcinoma, undifferentiated carcinoma, and adenosquamous carcinoma. NA: Not available

Table S2. The basic information of 80 patients with benign colorectal lesions for ALKAL1 immunohistochemical staining analysis.

		Cases (n)	Percentage (%)
Gender	Male	58	72.5
	Female	22	27.5
Age	<60	47	58.8
	≥60	33	41.3
Type of diseases	Adenoma	40	50.0
	Inflammation	9	11.3
	Polyp	25	31.3
	Other	6	7.5

* Adenoma includes tubular adenoma and villioustublar adenoma. Inflammation includes ulceration, chronic inflammation and abscess. Polyp includes adenomatous polyp, inflammatory polyp, inflammatory hyperplastic polyp, juvenile polyp and hyperplastic polyp. Other includes multiple diverticulum and congenital megacolon.

Table S3. The basic information of 20 patients with colorectal precancerous lesions for ALKAL1 immunohistochemical staining analysis.

		Cases (n)	Percentage (%)
Gender	Male	12	30.0
	Female	8	20.0
Age	<60	7	17.5
	≥60	12	30.0
	NA	1	2.5
Histologic	Adenomatous polyp with IN	3	7.5
	Tubular adenoma with IN	8	20.0
	Villioustublar adenoma with IN	9	22.5

* NA: Not available; IN: intraepithelial neoplasia.

Table S4. The basic information of 10 colorectal cancer patients for ALKAL1 mRNA and protein expression analysis.

		Cases (n)	Percentage (%)
Location	Colon	5	50.0
	Rectum	5	50.0
Gender	Male	5	50.0
	Female	5	50.0
Age	≤60	4	40.0
	>60	6	60.0
Grade	G1	1	10.0
	G2	8	80.0
	G3	1	10.0
Stage	Stage I	1	10.0
	Stage II	5	50.0
	Stage III	4	40.0
	Stage IV	0	0.0

Table S5. A list of primers used in the reactions for real-time RT-PCR.

Gene name		Sequence
GAPDH	forward	5'- GCACCGTCAAGGCTGAGAAC -3'
	reverse	5'- TGGTGAAGACGCCAGTGGA -3'
ACTB	forward	5'- TGGCACCCAGCACAATGAA -3'
	reverse	5'- CTAAGTCATAGTCCGCCTAGAAGCA -3'
ALKAL1	forward	5'- AGCAAACATTTCCACCGACT -3'
	reverse	5'- GCTAGGTCTGGGAGCACAGT -3'
MMP2	forward	5'- AACTACGATGATGACCGCAAG -3'
	reverse	5'- GACAGACGGAAGTTCTTGGTG -3'
MMP3	forward	5'- TACAAGGAGGCAGGCAAGAC -3'
	reverse	5'- GGATAGGCTGAGCAAACTGC-3'
MMP7	forward	5'- TGTATGGGGAACTGCTGACA-3'
	reverse	5'- ATCTCCTCCGAGACCTGTCC-3'
MMP9	forward	5'- CAGTCCACCCTTGTGCTCTT -3'
	reverse	5'- ATTTCGACTCTCCACGCATC -3'
TIMP1	forward	5'- TTTCTTGCTCCCTCTGGCTA -3'
	reverse	5'- AAAGGGAGCAGCCAGAATTT -3'
TIMP2	forward	5'- GGAAGTGGACTCTGGAAACG -3'
	reverse	5'- GGGGGCCGTGTAGATAAACT -3'
TIMP3	forward	5'- CTGACAGGTCGCGTCTATGA -3'
	reverse	5'- TGCAGTTACAACCCAGGTGA -3'
TIMP4	forward	5'- CTTGGTGCAGAGGGAAAGTC -3'
	reverse	5'- GTCCAGAGGCACTCGTTAGG -3'
CFL1	forward	5'- GCAAGAAGGAGGATCTGGTG -3'
	reverse	5'- GCTTGATCCCTGTCAGCTTC -3'
CFL2	forward	5'- ACGTCCAAACCCCTTTAAGAAG -3'
	reverse	5'- CTCCAGAAGCCATGTAAGTC-3'
HSP90AA1	forward	5'- GCCTCTGGTGATGAGATGGT-3'
	reverse	5'- ACGTTCCACAAAGGCTGAGT-3'
LIMK1	forward	5'- GGAGAGGAAGGAAGCGAGTT-3'
	reverse	5'- GCAGTCACAACACCTGAAGC-3'
LIMK2	forward	5'- ATGCACATCAGTCCCAACAA -3'
	reverse	5'- CGTCTGGCTAATTGCATCCT -3'
TESK1	forward	5'- CGTGTGGACGATTTTCACTG -3'
	reverse	5'- TGGGGAGCTTGTTCATCTTC -3'

PDXP	forward	5'- CGACCCCGAGTGCCTACT -3'
	reverse	5'- CGAGGCTGTCTCCACTGC -3'
ROCK1	forward	5'- GCACCAGTTGTACCCGATTT-3'
	reverse	5'- AGTTGATTGCCAACGAAAGC-3'
ROCK2	forward	5'- TGGGGTGGAAGAAATCAGAC -3'
	reverse	5'- TCATCGAAATTGCTGCTGTC -3'
PTCH	forward	5'- GCCATGGTTCTGCTCATTTT -3'
	reverse	5'- CCTGAATCACTCTGCTGACG -3'
HIP1	forward	5'- GCAGGAAGTGGCTGTAAAGG -3'
	reverse	5'- GCACTGCGTTGCTAGACAGA -3'
CCND1	forward	5'- CGTGGCCTCTAAGATGAAGG -3'
	reverse	5'- CCACTTGAGCTTGTTCACCA -3'
CCNE2	forward	5'- GGGGATCAGTCCTTGCATTA -3'
	reverse	5'- TCAGGCAAAGGTGAAGGATT-3'
HDAC1	forward	5'- TGGAAATCTATCGCCCTCAC -3'
	reverse	5'- CTGCTTGCTGTACTCCGACA -3'

Table S6. The basic information of 377 patients with colorectal cancer for ALKAL1 immunohistochemical staining analysis.

		Cases (n)	Percentage (%)
Location	Rectum	187	49.6
	Colon	178	47.2
	NA	12	3.2
Gender	Female	158	41.9
	Male	218	57.8
	NA	1	0.3
Age	<60	187	49.6
	⩾60	190	50.4
Histologic type	Non-specific[a]	316	83.8
	Other[b]	61	16.2
Grade	G1	28	7.4
	G2	253	67.1
	G3	27	7.2
	NA	69	18.3
T classification	T1	16	4.2
	T2	68	18.0
	T3	255	67.6
	T4	11	2.9
	NA	27	7.3
N classification	N0	199	52.8
	N1	91	24.1
	N2	63	16.7
	NA	24	6.4
M classification	M0	298	79.0
	M1	79	21.0
Stage	Stage I	56	14.9

Stage II	113	30.0
Stage III	108	28.6
Stage IV	79	21.0
NA	21	5.5

Noted: a) Non-specific type of adenocarcinoma included polypoid adenocarcinoma, tubular adenocarcinoma, and mucious adenocarcinoma. b) Other type included signet ring cell carcinoma, undifferentiated carcinoma, and adenosquamous carcinoma. NA: Not available

Table S7. The relationship between ALKAL1 IHC expression level and clinical pathological characteristics in 377 patients with colorectal cancer.

Parameters	Number of cases	ALKAL1 IHC expression		P values
		Low	High	
Location				
Rectum	187	92	95	0.1162
Colon	178	73	105	
Gender				
Female	158	77	81	0.2425
Male	218	93	125	
Age				
<60	187	92	95	0.1120
⩾60	190	78	112	
Histologic type				
Non-specific[a]	316	146	170	0.3243
Other[b]	61	24	37	
Grade				
G1-G2	281	134	147	0.1532
G3	27	9	18	
T classification				
T1-2	84	41	43	0.4753
T3-4	266	118	148	
N classification				
N0	199	100	99	0.0465
N1-2	154	61	93	
M classification				
M0	298	147	151	0.0013
M1	79	23	56	

Stage				
I-II	169	92	77	0.0006
III-IV	187	68	119	

Noted: a) Non-specific type of adenocarcinoma included polypoid adenocarcinoma, tubular adenocarcinoma, and mucious adenocarcinoma. b) Other type included signet ring cell carcinoma, undifferentiated carcinoma, and adenosquamous carcinoma.
